# “Pure” hepatoid tumors of the pancreas harboring *CTNNB1* somatic mutations: a new entity among solid pseudopapillary neoplasms

**DOI:** 10.1007/s00428-022-03317-4

**Published:** 2022-03-31

**Authors:** Paola Mattiolo, Andrea Mafficini, Rita T. Lawlor, Giovanni Marchegiani, Giuseppe Malleo, Antonio Pea, Roberto Salvia, Paola Piccoli, Concetta Sciammarella, Nicola Santonicco, Alice Parisi, Nicola Silvestris, Michele Milella, Volkan Adsay, Aldo Scarpa, Claudio Luchini

**Affiliations:** 1 Department of Diagnostics and Public Health, Section of Pathology, University and Hospital Trust of Verona, Piazzale Scuro, 10, 37134 Verona, Italy; 2 ARC-Net Research Center for Applied Research On Cancer, University of Verona, 37134 Verona, Italy; 3 Department of Surgery, The Pancreas Institute, University and Hospital Trust of Verona, 37134 Verona, Italy; 4 Medical Oncology Unit, Department of Human Pathology “G. Barresi”, University of Messina, 98125 Messina, Italy; 5 Department of Medicine, Section of Medical Oncology, University and Hospital Trust of Verona, 37134 Verona, Italy; 6 Department of Pathology, Koç University Hospital and Koç University Research Center for Translational Medicine (KUTTAM), 34010 Istanbul, Turkey

**Keywords:** Hepatoid, Pancreas, Pancreatic, Pancreatic ductal adenocarcinoma, PDAC, Hep Par-1, *CTNNB1*, Solid pseudopapillary

## Abstract

Hepatoid tumors (HTs) represent a rare group of neoplasms that are histologically similar to hepatocellular carcinoma but arise outside the liver. The current World Health Organization classification recognizes the hepatoid morphology of pancreatic tumors only as a possible variant of pancreatic ductal adenocarcinoma (PDAC). Here, we describe two cases of “pure” HT of the pancreas showing common features and characterized by indolent biological behavior. These tumors were roundish nodules with pushing borders, hyaline globules, and pure hepatoid histology; they were diffusely positive for β-catenin and LEF1 on immunohistochemistry. At next-generation sequencing, both neoplasms harbored only one pathogenic somatic mutation that affected the *CTNNB1* gene at exon 3 and showed a loss of heterozygosity on chromosomes 18 and 21. By integrating macroscopic and microscopic features, along with their molecular profiles, we advocate that such tumors represent a distinct entity from PDAC and should be considered a new variant of solid pseudopapillary neoplasms. The recognition of this new neoplastic category may have immediate implications not only for tumor taxonomy but also for clinical practice.

**Supplementary Information:**

The online version contains supplementary material available at 10.1007/s00428-022-03317-4.

## Introduction

Tumors with hepatoid differentiation represent a rare group of cancers that are histologically similar to hepatocellular carcinoma but arise outside the liver [1–4]. This tumor type usually shows a solid or solid-trabecular architecture and is composed of polyhedral cells with large and eosinophilic cytoplasm, central nuclei, and evident nucleoli [1–5]. The biological behavior of hepatoid tumors (HTs) is still not well understood mainly because of their rarity; however, most reports indicate aggressive behavior with early metastasis [1–3].

Although morphology is the only acknowledged criterion for diagnosis, positivity for some immunohistochemical markers such as hepatocyte paraffin-1 (Hep Par-1), CD10, alpha-fetoprotein, and arginase-1 may be helpful in supporting the identification of hepatoid differentiation [1, 6–8].

A recent study investigating with next-generation sequencing the genomic profile of HT from different organs did not reveal any common molecular hallmarks to explain the peculiar hepatoid morphology and concluded that the tissue of origin is the most important factor influencing their molecular landscape [5]. Therefore, the tissue of origin and genomic profile may be important in influencing tumor morphology and the expression of hepatocyte markers.

Regarding pancreatic HT, the current World Health Organization (WHO) classification officially recognizes hepatoid morphology as a possible variant of pancreatic ductal adenocarcinoma (PDAC), named hepatoid carcinoma [1, 9–13]. Regarding other pancreatic tumors, neuroendocrine neoplasms can also show variable degrees of hepatoid differentiation [14–16], whereas intraductal oncocytic papillary neoplasms are typically positive for the hepatoid marker Hep Par-1 [17, 18].

Here, we describe two cases of pure HT of the pancreas showing the same morphological, immunohistochemical, and molecular profiles and characterized by indolent biological behavior. These tumors cannot be considered either PDAC or a neuroendocrine variant, thus representing a new potential entity among pancreatic tumors.

## Materials and methods

Two cases of “pure” HT of the pancreas, showing 100% hepatoid morphology, have been identified in the pathology archives of the University and Hospital Trust of Verona (ARC-Net Biobank). All clinicopathological parameters were recorded, including the clinical history updated at the time of the last follow-up.

### Immunohistochemistry (IHC)

The cases were investigated using IHC at the time of diagnosis. However, in this study, IHC was repeated with the addition of other markers. IHC was performed as previously described [19], according to the manufacturer’s instructions, and evaluated blindly by two pancreatic pathologists (C.L. and A.S.).

Globally considered, the following antibodies were used: alpha-fetoprotein (polyclonal/rabbit, dilution: 1:300, source: Dako/Germany), androgen receptor (AR411, 1:20, Dako), arginase-1 (clone: SP156, 1:100, Cell Marque/USA), CD10 (56C6, 1:50, Novocastra/UK), BAP1 (C-4, 1:100, Santa Cruz Biotechnology/Germany), CD56 (BC56C04, 1:150, Biocare/USA), CD117 (EP10, pre-diluted, Leica/Italy), CD200 (goat, 1:200, RD Systems/USA), cytokeratin 7 (RN7, 1:100, Novocastra), cytokeratin 8/18/19 (5D3, pre-diluted, Leica), cytokeratin 20 (PW31, 1:100, Novocastra), cytokeratin AE1/AE3 (AE1-AE3, pre-diluted, Novocastra), β-catenin (15B8, 1:400, Sigma Aldrich/USA), BCL10 (331.3, 1:1000, Santa Cruz Biotechnology), chromogranin-A (DAK-A3, 1:2500, Dako), E-cadherin (NCH-38, 1:20, Dako), Hep Par-1 (OCH1E5, 1:50, Dako), KDM6A (D3Q11, 1:200, Cell Signalling Technology/The Netherlands), LEF-1 (EPR2023Y, 1:200, Novus-Abcam/UK), MUC1 (MA695, pre-diluted, Leica), MUC2 (CCP58, pre-diluted, Novocastra), MUC5AC (CLH2, 1:50, Dako), MUC6 (CLH5, 1:100, Abnova/Taiwan), progesterone receptor (PgR636, 1:150, Dako), SMAD4 (B-8, 1:1000, Santa Cruz Biotechnology), synaptophysin (27G12, pre-diluted, Novocastra), trypsin (rabbit, 1:500, Tema/Italy), and vimentin (V9, 1:50, Novocastra).

### Massive parallel sequencing (next-generation sequencing, NGS)

DNA extracted from formalin-fixed paraffin-embedded tissues was subjected to NGS using the SureSelectXT HS CD Glasgow Cancer Core assay (www.agilent.com), hereafter referred to as CORE, as previously described [20, 21]. The panel spans 1.85 megabases of the genome and interrogates 174 genes for somatic mutations, copy number alterations, and structural rearrangements; the details of the targeted genes are reported in Supplementary Table 1. Sequencing libraries were prepared by targeted capture using the SureSelect kit (Agilent Technologies) with RNA baits targeting a bespoke set of selected genomic features. Sequencing was performed on a NextSeq 500 (Illumina) loaded with two captured library pools using a high-output flow cell and 2 × 75 bp paired-end sequencing.

CORE panel analysis started with demultiplexing was performed with FASTQ Generation v1.0.0 on the BaseSpace Sequence Hub (https://basespace.illumina.com, last access 03/23/2021). Forward and reverse reads from each demultiplexed sample were aligned to the human reference genome (version hg38/GRCh38) using BWA and saved in the BAM file format.

Single-nucleotide variants were identified using Shearwater [22]. Small (< 200 bp) insertions and deletions were called using Pindel [23]. Small nucleotide variants were further annotated using a custom pipeline based on vcflib (https://github.com/ekg/vcflib; last access 11/30/2020), SnpSift [24], Variant Effect Predictor software [25], and the NCBI RefSeq transcripts database (https://www.ncbi.nlm.nih.gov/refseq/; last access 11/30/2020). All candidate mutations were manually reviewed using Integrative Genomics Viewer (IGV) version 2.9 [26] to exclude sequencing artifacts.

Tumor mutational burden and microsatellite instability were derived from sequencing analysis and computed according to the method described by Papke et al. [27]. Copy number alterations of targeted genes were detected using geneCN software, developed at Wolfson Wohl Cancer Research Centre (https://github.com/wwcrc/geneCN; last access 10/31/2020). Structural rearrangements were detected using BRASS software [28] and visually reviewed using IGV, version 2.9, to exclude sequencing artifacts.

### Variant classification

Variants were classified according to the five-tier classification system recommended by the joint consensus of the American College of Medical Genetics and Genomics and the Association for Molecular Pathology (ACMG/AMP) [29]. Variants were thus classified as benign (class 1), likely benign (class 2), variants of uncertain significance (VUS—class 3), likely pathogenic (class 4), and pathogenic (class 5).

## Results

### Clinicopathological and histological features

**Fig. 1:**
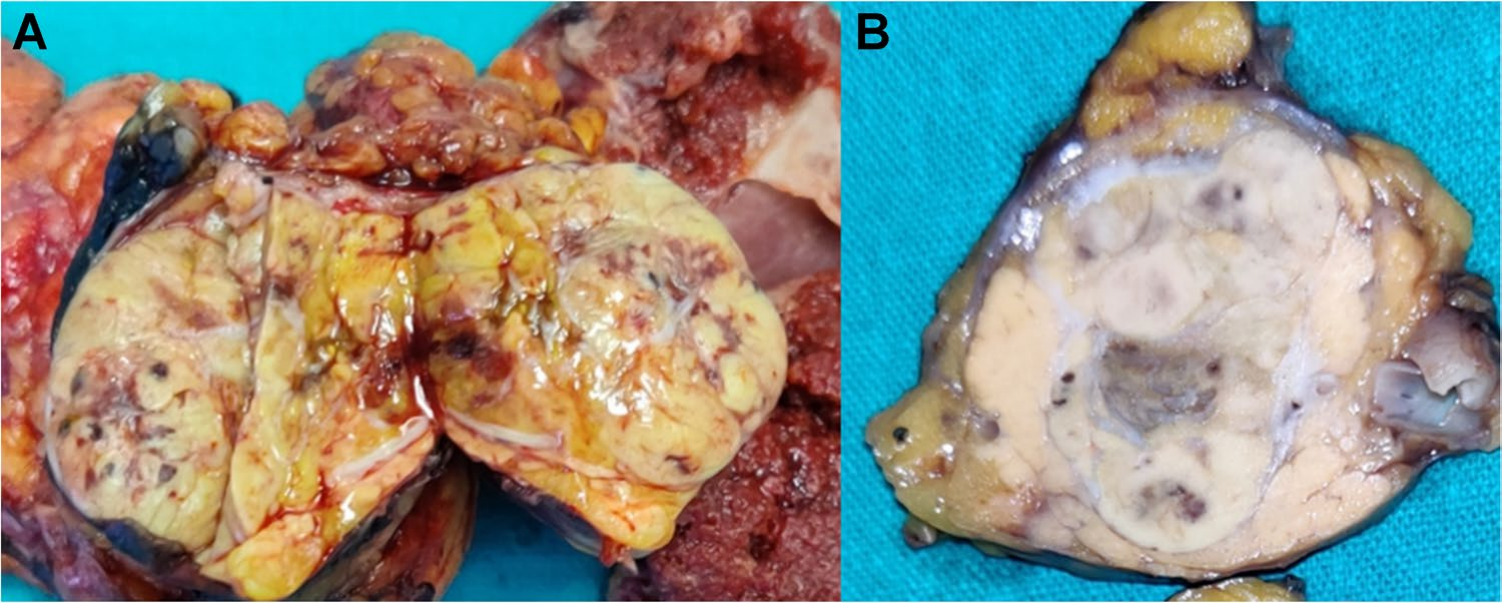
Macroscopic image of case #2. Tumor mass was located in the pancreatic tail and appeared as a round yellow-brownish nodule, with pushing borders. Also case #1 had the same features, but arose in the pancreatic head

Both patients were male; the first patient was 53 years old and the second was 56 years old at the time of diagnosis. Both tumors were roundish with pushing borders and yellow-brownish at grossing (Fig. 1), involving the pancreatic head in the first patient and the pancreatic tail in the second. The main axis of the tumor at the pancreatic head was 5 cm, whereas that of the tumor at the tail was 3.7 cm. In both cases, the clinical presentation was undefined abdominal pain, and no liver lesions were detected on imaging.

**Fig. 2:**
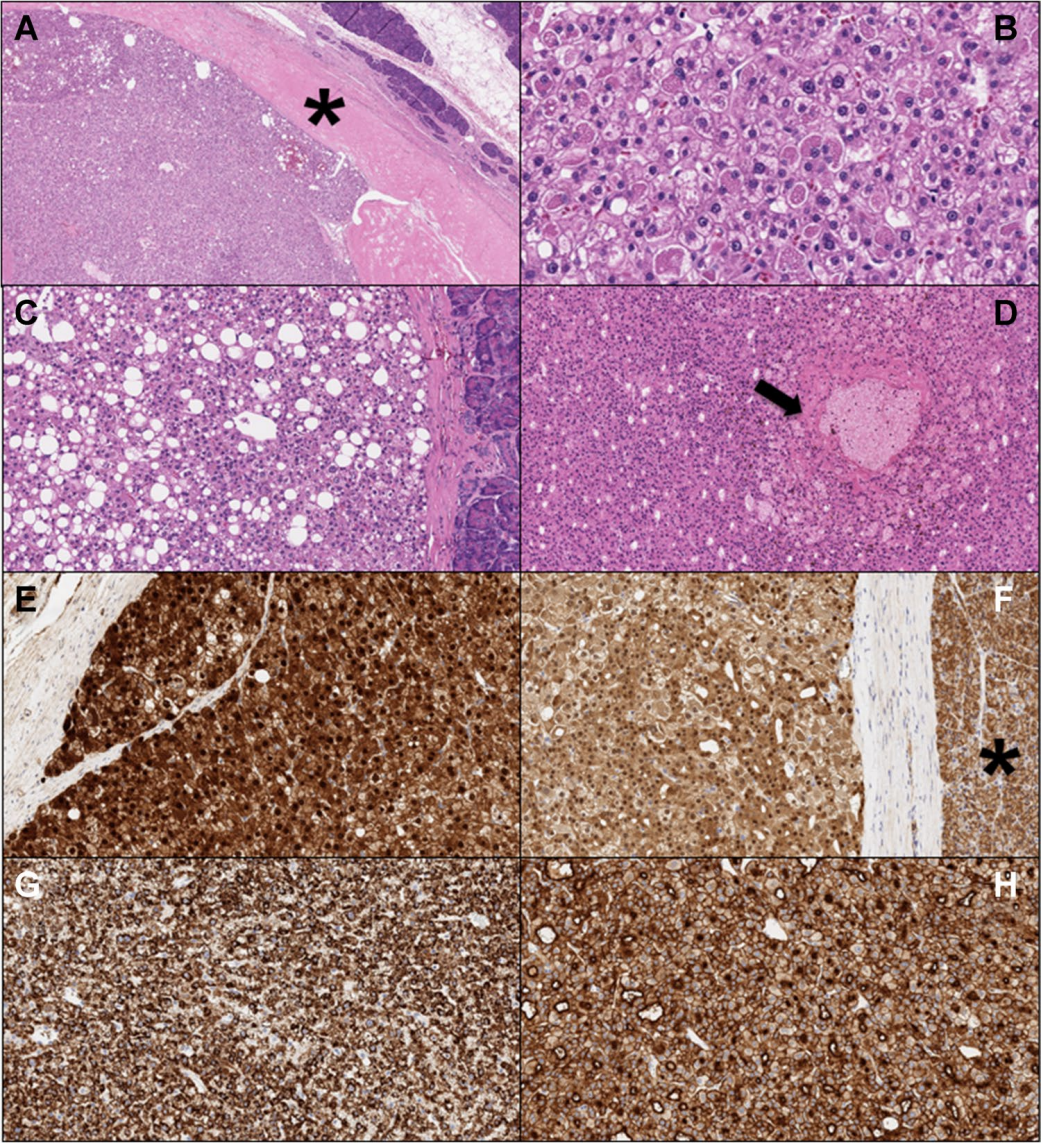
Highly illustrative microscopic images of the described cases: **A** low-magnification image showing tumor pushing borders and the thickened tumor capsule (hematoxylin–eosin, 4 × original magnification; tumor capsule is indicated with a black asterisk); **B** both tumors show the focal presence of hyaline globules (hematoxylin–eosin, 20 × original magnification); **C** some areas with “steatohepatitis-like” appearance are also present (hematoxylin–eosin, 10 × original magnification); **D** foci of foamy macrophages are encountered (black arrow; hematoxylin–eosin, 10 × original magnification); **E**, **F** beta-catenin nuclear positivity in both cases (**E** 20 × , **F** 10 × original magnification; in **F**, the normal pancreatic parenchyma with normal membranous staining pattern is indicated with a black asterisk); **G** Hep Par-1 demonstrated a strong and diffuse staining pattern (10 × original magnification); **H** CD10 demonstrated a strong staining pattern, with a typical canalicular enhancement (10 × original magnification)

On histological assessment (Fig. 2), both neoplasms showed the presence of a tumor capsule, and the margins did not show any infiltrative growth toward the pancreatic parenchyma. The neoplasms were composed of large cells with eosinophilic cytoplasm, central nuclei, and prominent nucleoli. The architecture was solid and solid-trabecular. Thus, both tumors had a typical “hepatoid” appearance. The neoplasms also displayed “steatohepatitis-like” areas (more pronounced in patient number 2) and foamy-macrophage aggregates, and had focally reached hyaline globules. Vascular and perineural infiltrations were lacking. No nodal metastases were observed in either case. By applying the current TNM staging system, the first neoplasm was staged as pT3N0M0 and the second as pT2N0M0.

Both patients underwent surgical resection for suspected neuroendocrine tumors. The first patient, who underwent pancreaticoduodenectomy in 2009, did not report any relapse during follow-up: after 12 years and 4 months, the patient was alive and still completely free of disease. The second patient underwent distal pancreatectomy in 2021, and was free of disease after 10 months.

### Immunohistochemical profile

Both tumors displayed the same IHC profiles. The tumor cells were positive for arginase-1, CD10 (with canalicular enhancement), CD56, cytokeratin 8/18/19, Hep Par-1, β-catenin, LEF1, and androgen receptor (weak positivity). At the same time, they were negative for the neuroendocrine markers chromogranin-A and synaptophysin; for different types of cytokeratin such as cytokeratin 7, cytokeratin 20, and cytokeratin AE1/AE3; for the acinar markers BCL10 and trypsin; for some solid pseudopapillary markers such as CD200, progesterone receptor, and vimentin; for the following mucins: MUC1, MUC2, MUC5AC, and MUC6; and for alpha-fetoprotein, SMAD4, and CD117. Both neoplasms showed a loss of E-cadherin and conserved expression of KDM6A; BAP1 showed a heterogeneous staining pattern in the first tumor and conserved expression in the second neoplasm.

### Molecular profile

Case number 1 was sequenced to a median coverage depth of 123 × and the tumor cellularity was 90%. Case number 2 had a median coverage depth of 139 × , while the tumor cellularity was 75%. In NGS, both cases harbored only one pathogenic somatic mutation affecting exon 3 of the *CTNNB1* gene. The mutation in the first case was c.102_113del, resulting in an in-frame deletion (p.I35_G38del, VAF = 50%), and the mutation in the second case was c.109 T > G, resulting in a missense substitution (p.S37A, VAF = 37%). Both cases were microsatellite-stable; the first case presented a tumor mutational burden of 4.86 mut/Mb, and the second case presented that of 9.19 mut/Mb. Additional VUS were detected for each case (Supplementary Table 2).

Copy number variation analysis revealed loss of heterozygosity (LOH) on chromosomes 18 and 21 in both cases, gain of heterozygosity on chromosome 20 in patient 1, and LOH on chromosome 1 (region of 1p36.33-p12) in patient 2 (Supplementary Table 2).

## Discussion

In this manuscript, two pancreatic neoplasms with common macroscopic, histopathological, and molecular features are reported. An integrated histological and molecular approach has allowed the identification of a new potential entity among pancreatic tumors.

Currently, the WHO classification officially recognizes hepatoid morphology as a possible PDAC variant [1]. Notably, such variants are also associated with aggressive biological behavior, with a high rate of vascular invasion and early metastasis. Intriguingly, the present report documents the potential existence of a new entity among pancreatic neoplasms with hepatoid morphology. The two reported cases showed distinct features at different levels of analysis.

Macroscopically, the tumors were yellow-brownish nodules with well-demarcated and pushing borders that were limited to the surrounding pancreatic parenchyma by a distinct capsule. Conversely, the only HT entity with an already WHO-recognized presence, which is considered a PDAC variant, is usually whitish with infiltrative margins. Some of the reported HTs in the biliopancreatic region have shown a roundish appearance, but they have also shown infiltrative margins or growth [5, 10, 14], which was not present in the cases described here. The tumor capsule is a potentially important histological parameter in pancreatic neoplasms, such as solid pseudopapillary neoplasms (SPNs) and well-differentiated pancreatic neuroendocrine tumors [30, 31], and the lack of capsule infiltration by neoplastic cells is in line with indolent biological behavior. In a recent investigation by Lee et al. on 375 surgically resected SPNs, the authors considered lymphovascular invasion, perineural invasion, synchronous or metachronous metastasis, and adjacent organ invasion as malignant histopathological features [32]. Of note, both tumors in this study lacked the aforementioned microscopic features. This may further confirm their indolent nature, which is extremely different from that of PDAC.

Histologically, both tumors were hypercellular and were composed of large eosinophilic elements with a typical hepatoid appearance, resembling a true hepatocellular carcinoma. In contrast to the classical presentation of PDAC, vascular invasion, perineural infiltration, and nodal metastases were lacking, supporting a low malignant potential. Interestingly, both tumors were focally rich in hyaline globules, which are microscopic features already described in pancreatic SPNs and, less frequently, in well-differentiated neuroendocrine tumors [33, 34]. Notably, the focal presence of aggregates of foamy macrophages is also described as a common histological finding encountered in SPN [34].

From an immunohistochemical point of view, the two reported neoplasms expressed the hepatoid markers arginase-1, CD10, and Hep Par-1, and were also positive for some markers typically expressed in tumor types other than PDAC, such as β-catenin and LEF-1, classically positive and very specific for SPN [35, 36], and CD56, usually positive in neuroendocrine tumors, although without a high specificity. This IHC expression pattern further corroborates the fact that the reported cases represent a distinct entity from PDAC. Of note, both neoplasms were negative for the classic neuroendocrine markers chromogranin-A and synaptophysin; for different types of cytokeratin, such as cytokeratin 7, cytokeratin 20, and cytokeratin AE1/AE3; for the acinar markers BCL10 and trypsin; and for some solid pseudopapillary markers such as CD200, progesterone receptor, and vimentin. The lack of expression of these markers further highlights the peculiarity of the two described tumors, supporting their classification as distinct entities among pancreatic tumors. Interestingly, at the IHC level and based on CD10, β-catenin, and LEF-1 positivity, the tumor entity closest to the reported tumors was pancreatic SPN.

Along this line, the molecular profile is even more significant. Notably, the integration of histomorphology and IHC with genomic characterization seemed to represent a decisive step in understanding the real nature of the reported neoplasms. Both tumors displayed only one pathogenic somatic mutation, which was *CTNNB1* mutation. This type of molecular alteration definitively supports the distinction from PDAC. Furthermore, it suggests that both neoplasms could be considered within the SPN-spectrum. The molecular hallmark of SPN is point mutations in exon 3 of *CTNNB1* [34, 37]. Notably, in the reported cases, the mutations involved the same exon of *CTNNB1*. Mutations affecting the same gene and the nuclear β-catenin staining pattern may be detected in other types of pancreatic neoplasms, but are more rarely observed, being present in < 10% of acinar cell carcinomas and in < 1% of neuroendocrine tumors [1, 38–40]. Of note, the presence of only one pathogenic mutation in the reported cases further corroborates the likelihood of their “SPN-nature,” because SPNs harbor very few mutations compared with other solid pancreatic malignancies [41]. Another interesting finding was copy number variation analysis. Indeed, both tumors harbored LOH on chromosomes 18 and 21, and LOH on chromosome 21 is another molecular alteration previously described in SPNs [42].

In conclusion, through the report of two parallel cases, we describe the emergence of a potential new tumor entity among pancreatic neoplasms. The integration of macroscopic and microscopic data, along with their molecular profiles, indicates that pancreatic tumors with a “hepatoid” morphology can also exist outside the PDAC spectrum, in contrast to the current WHO classification. Moreover, based on our integrative analysis, we advocate that pancreatic roundish tumors without nodal metastasis and with hyaline globules, hepatoid morphology, β-catenin and LEF1 positivity on IHC, *CTNNB1* mutation on exon 3, and LOH on chromosome 21 on NGS should be considered as a new variant of pancreatic SPN. The recognition of this new neoplastic category may have immediate implications not only for tumor classification but also for clinical practice.

## Supplementary Information

Below is the link to the electronic supplementary material.

Supplementary file1 (PDF 43 KB)

Supplementary file2 (DOCX 16 KB)

## Data Availability

All data/information are available in the manuscript and in the supplementary material.
