# Supplementary material for: “Pure” hepatoid tumors of the pancreas harboring CTNNB1 somatic mutations: a new entity among solid pseudopapillary neoplasms
Source: Virchows Arch. 2022 Mar 31;481(1):41–7. doi: 10.1007/s00428-022-03317-4 (PMC9226109; doi:10.1007/s00428-022-03317-4)
Supplement: Supplementary file 2 — Supplementary file2 (DOCX 16 KB) [file 428_2022_3317_MOESM2_ESM.docx]

**Supplementary Table 2:** TMB, CNV and variants of uncertain significance in the reported cases

| **ID case** | **TMB** | **Gene Alterations** | | | | | **CNV** | |
| --- | --- | --- | --- | --- | --- | --- | --- | --- |
|  |  | **Gene** | **Variation** | **Mutation type** | **Freq** | **Class*** | **Site** | **Variation** |
| **Case #1** | 4.86 | *PDGFRB* | p.P1082L | Substitution – Missense | 39 | 3 | chr18  chr21  chr20 | LOH  LOH  gain |
| **Case #2** | 9.19 | *APC* | p.G1721R | Substitution – Missense | 45 | 3 | chr1p36.33-p12 | LOH |
|  |  | *CREBBP* | p.A2265V | Substitution – Missense | 48 | 3 | chr18 | LOH |
|  |  | *ERBB2* | p.R896H | Substitution – Missense | 45 | 3 | chr21 | LOH |
|  |  | *ERBB3* | p.P514_G515delinsIS | Substitution – Missense | 38 | 3 |  |  |
|  |  | *MED12* | p.Y299N | Substitution – Missense | 79 | 3 |  |  |
|  |  | *NOTCH3* | p.Y1197H | Substitution – Missense | 50 | 3 |  |  |
|  |  | *PDCD1LG2* | p.P81S | Substitution – Missense | 42 | 3 |  |  |
|  |  | *SYK* | p.R625W | Substitution – Missense | 45 | 3 |  |  |

Abbreviations: TMB: tumor mutational burden; CNV: copy number variation; LOH: loss of heterozygosity.
